# Supplementary material for: The association of D-dimer with clinicopathological features of breast cancer and its usefulness in differential diagnosis: A systematic review and meta-analysis
Source: PLoS One. 2019 Sep 5;14(9):e0221374. doi: 10.1371/journal.pone.0221374 (PMC6728019; doi:10.1371/journal.pone.0221374)
Supplement: S1 Table — (DOCX) [file pone.0221374.s001.docx]

| **Database** |  |  | **Search terms** |
| --- | --- | --- | --- |
| **PUBMED** |  |  | **Breast Cancer** |
|  |  | 1 | Breast Neoplasms [Mesh] |
|  |  | 2 | Carcinoma*, Breast[Title/Abstract] |
|  |  | 3 | Breast Carcinoma*[Title/Abstract]) |
|  |  | 4 | Mammary Neoplasm, Human[Title/Abstract] |
|  |  | 5 | Neoplasm*, Human Mammary[Title/Abstract] |
|  |  | 6 | Mammary Neoplasms, Human[Title/Abstract] |
|  |  | 7 | Human Mammary Neoplasm*[Title/Abstract] |
|  |  | 8 | Human Mammary Carcinoma[Title/Abstract] |
|  |  | 9 | Human Mammary Carcinomas[Title/Abstract] |
|  |  | 10 | Mammary Carcinomas, Human[Title/Abstract] |
|  |  | 11 | Carcinoma*, Human Mammary[Title/Abstract] |
|  |  | 12 | Mammary Carcinoma, Human[Title/Abstract] |
|  |  | 13 | Cancer of the Breast[Title/Abstract] |
|  |  | 14 | Cancer of Breast[Title/Abstract] |
|  |  | 15 | Breast Malignant Tumor*[Title/Abstract] |
|  |  | 16 | Malignant Tumor of Breast[Title/Abstract] |
|  |  | 17 | Breast Malignant Neoplasm*[Title/Abstract] |
|  |  | 18 | Malignant Neoplasm of Breast[Title/Abstract]) |
|  |  | 19 | Mammary Cancer*[Title/Abstract]) |
|  |  | 20 | Cancer*, Mammary[Title/Abstract] |
|  |  | 21 | Cancer, Breast[Title/Abstract] |
|  |  | 22 | Breast Cancer[Title/Abstract] |
|  |  | 23 | Tumor*, Breast[Title/Abstract] |
|  |  | 24 | Neoplasms, Breast[Title/Abstract] |
|  |  | 25 | Breast Tumor*[Title/Abstract] |
|  |  | 26 | Neoplasm, Breast[Title/Abstract] |
|  |  | 27 | Breast Neoplasm[Title/Abstract] |
|  |  | 28 | or/1-28 |
|  |  |  | **D-Dimer** |
|  |  | 29 | fibrin fragment D [Supplementary Concept] |
|  |  | 30 | D-dimer fibrin[Title/Abstract] |
|  |  | 31 | D-dimer[Title/Abstract] |
|  |  | 32 | fibrin fragment DD[Title/Abstract] |
|  |  | 33 | fibrin fragment D-dimer[Title/Abstract] |
|  |  | 34 | fibrin fragment D1 dimer[Title/Abstract] |
|  |  | 35 | D-dimer fragments[Title/Abstract] |
|  |  | 36 | or/29-35 |
|  |  |  | **Combination** |
|  |  | 37 | 28 and 36 |
| **EMBASE** |  |  | **Breast Cancer** |
|  |  | 1 | 'breast cancer'/exp |
|  |  | 2 | 'carcinoma*, breast':ti,ab |
|  |  | 3 | ' Breast Carcinoma* ':ti,ab |
|  |  | 4 | ' Mammary Neoplasm, Human ':ti,ab |
|  |  | 5 | ' Neoplasm*, Human Mammary ':ti,ab |
|  |  | 6 | 'Mammary Neoplasms, Humany ':ti,ab |
|  |  | 7 | ' Human Mammary Neoplasm* ':ti,ab |
|  |  | 8 | ' Human Mammary Carcinoma ':ti,ab |
|  |  | 9 | ' Human Mammary Carcinomas':ti,ab |
|  |  | 10 | ' Mammary Carcinomas, Human ':ti,ab |
|  |  | 11 | ' Carcinoma*, Human Mammary ':ti,ab |
|  |  | 12 | ' Mammary Carcinoma, Human ':ti,ab |
|  |  | 13 | ' Cancer of the Breast ':ti,ab |
|  |  | 14 | ' Cancer of Breast ':ti,ab |
|  |  | 15 | ' Breast Malignant Tumor* ':ti,ab |
|  |  | 16 | ' Malignant Tumor of Breast ':ti,ab |
|  |  | 17 | ' Breast Malignant Neoplasm* ':ti,ab |
|  |  | 18 | ' Malignant Neoplasm of Breast ':ti,ab |
|  |  | 19 | ' Mammary Cancer* ':ti,ab |
|  |  | 20 | ' Cancer*, Mammary ':ti,ab |
| **COCHRANE LIBRARY** |  | 21 | ' Cancer, Breast ':ti,ab |
|  |  | 22 | ' Breast Cancer ':ti,ab |
|  |  | 23 | ' Tumor*, Breast ':ti,ab |
|  |  | 24 | ' Neoplasms, Breast ':ti,ab |
|  |  | 25 | ' Breast Tumor* ':ti,ab |
|  |  | 26 | ' Neoplasm, Breast ':ti,ab |
|  |  | 27 | ' Breast Neoplasm ':ti,ab |
|  |  | 28 | or/1-28 |
|  |  |  | **D-Dimer** |
|  |  | 29 | ' fibrin fragment D ':ti,ab |
|  |  | 30 | ' D-dimer fibrin ':ti,ab |
|  |  | 31 | ' D-dimer ':ti,ab |
|  |  | 32 | ' f ibrin fragment DD ':ti,ab |
|  |  | 33 | ' fibrin fragment D-dimer ':ti,ab |
|  |  | 34 | ' fibrin fragment D1 dimer ':ti,ab |
|  |  | 35 | ' D-dimer fragments ':ti,ab |
|  |  | 36 | or/29-35 |
|  |  |  | **Combination** |
|  |  | 38 | 28 and 36 |
|  |  |  | **Breast Cancer** |
|  |  | 1 | MeSH descriptor: [Breast Neoplasms] explode all trees |
|  |  | 2 | (Breast Neoplasm):ti,ab,kw OR (Neoplasm, Breast):ti,ab,kw OR (Breast Tumor*):ti,ab,kw OR (Tumor*, Breast):ti,ab,kw OR (Neoplasms, Breast):ti,ab,kw (Word variations have been searched) |
|  |  | 3 | (Breast Cancer):ti,ab,kw OR (Cancer, Breast):ti,ab,kw OR (Mammary Cancer*):ti,ab,kw OR (Cancer*, Mammary):ti,ab,kw OR (Malignant Neoplasm of Breast):ti,ab,kw (Word variations have been searched) |
|  |  | 4 | (Breast Malignant Neoplasm*):ti,ab,kw OR (Malignant Tumor of Breast):ti,ab,kw OR (Breast Malignant Tumor*):ti,ab,kw OR (Cancer of Breast):ti,ab,kw OR (Cancer of the Breast):ti,ab,kw (Word variations have been searched) |
|  |  | 5 | (Mammary Carcinoma, Human):ti,ab,kw OR (Carcinoma*, Human Mammary):ti,ab,kw OR (Human Mammary Carcinomas):ti,ab,kw OR (Mammary Carcinomas, Human):ti,ab,kw OR (Human Mammary Carcinoma):ti,ab,kw (Word variations have been searched) |
|  |  | 6 | (Mammary Neoplasms, Human):ti,ab,kw OR (Human Mammary Neoplasm*):ti,ab,kw OR (Neoplasm*, Human Mammary):ti,ab,kw OR (Mammary Neoplasm, Human):ti,ab,kw OR (Breast Carcinoma*):ti,ab,kw (Word variations have been searched) |
|  |  | 7 | (Carcinoma*, Breast):ti,ab,kw (Word variations have been searched) |
|  |  | 8 | #1 OR #2 OR #3 OR #4 OR #5 OR #6 OR #7 |
|  |  |  | **D-Dimer** |
|  |  | 9 | (D-dimer fibrin):ti,ab,kw OR (D-dimer fragments):ti,ab,kw OR (fibrin fragment D1 dimer):ti,ab,kw OR (fibrin fragment DD):ti,ab,kw OR (D-dimer):ti,ab,kw (Word variations have been searched) |
|  |  | 10 | (fibrin fragment D-dimer):ti,ab,kw (Word variations have been searched) |
|  |  | 11 | #9 OR #10 |
|  |  |  | **Combination** |
|  |  | 12 | #8 AND #11 |
