# Supplementary material for: The association of D-dimer with clinicopathological features of breast cancer and its usefulness in differential diagnosis: A systematic review and meta-analysis
Source: PLoS One. 2019 Sep 5;14(9):e0221374. doi: 10.1371/journal.pone.0221374 (PMC6728019; doi:10.1371/journal.pone.0221374)
Supplement: Attachment — Submitted filename: Response_to_Reviewers.doc [file pone.0221374.s005.doc]

August 2, 2019

Dear Dr. Tagliabue:

We would like to thank you and the reviewers for your comments regarding our manuscript titled “The association of D-dimer with clinicopathological features of breast cancer and its usefulness in differential diagnosis: a systematic review and meta-analysis” (ID: PONE-D-19-14829R1).

Your comments have been helpful in revising and improving our manuscript. I have made the necessary minor revisions as per your suggestions.

Of the 15 articles included, ten were from China and five were from countries outside China. Because our meta-analysis is based on published research, the fact that most of the data coming from China may have been perceived as a problem. Considering that the five documents from countries other than China are also main components of the article, they are distributed in each group of the study, so specification of the results referring to the Chinese population in the title may lead to increased limitations of the meta-analysis.

We have adopted the reviewer's opinion that "The risk of regional bias should not be under evaluated". We have added Figure 4, using subgroup analysis to evaluate the impact of region on heterogeneity, and discussed this issue in the “Discussion”.

As follows, “The subgroup analysis was also used to examine the source of heterogeneity based on region with only significant differences in the benign control group. However, after excluding the study by Kim et al. of Korea from the benign control group, the heterogeneity between the eight Studies from China did not reduce, indicating that the regional differences cannot explain the heterogeneity between benign control groups."

We hope that this meta-analysis is not just another end point of research but that it encourages us to conduct more validation studies in other independent large groups, which will better clarify the association of D-dimer with breast cancer patients. This meta-analysis is limited, and we will continue to pay attention to the latest research in this area. When there is valuable literature, we will update it in time.

The minor revisions requested in the text are highlighted in yellow. The Figure 4 is given below. We hope that the manuscript is now acceptable for submission to PLOS ONE. We look forward to hearing from you.

Sincerely,

LY Zhang

Dongyang People’s Hospital

60 West Wuning Road, Dongyang 322100, Zhejiang, China

Tel: +8615267914600

Email: [Happy_zhang1y@163.com](mailto:Happy_zhang1y@163.com)

Fig 4. Subgroup analysis of D-dimer levels and breast cancer-associated differential diagnosis and clinicopathological features according to region.Plots depicting comparisons between breast cancer patients and (A) benign controls, (B) healthy controls, (C) tumor node metastasis (TNM) stage (stage III-IV vs stage I-II), (D) lymph node status (positive vs. negative), and (E)estrogen receptor (ER) status (positive vs. negative). Group1: China; Group2: Regions outside China
